# Correction: Normative reference values of handgrip strength for Brazilian older people aged 65 to 90 years: Evidence from the multicenter Fibra‑BR study

**DOI:** 10.1371/journal.pone.0265915

**Published:** 2022-03-17

**Authors:** Michael Eduardo Reichenheim, Roberto Alves Lourenço, Janaína Santos Nascimento, Virgílio Garcia Moreira, Anita Liberalesso Neri, Rodrigo Martins Ribeiro, Lygia Paccini Lustosa, Eduardo Ferriolli

## Notice of republication

This article was republished on March 10, 2022, because an incorrect version of the manuscript was mistakenly used in the original publication. The publisher apologizes for the errors. Please download this article again to view the correct version. The originally published, uncorrected article and the republished, corrected articles are provided here for reference.

## Supporting information

S1 File Originally published, uncorrected article. (PDF)

S2 File Republished, corrected article. (PDF)
