# Supplementary figures and images for: Normative reference values of handgrip strength for Brazilian older people aged 65 to 90 years: Evidence from the multicenter Fibra‑BR study
Source: PLoS One. 2021 May 4;16(5):e0250925. doi: 10.1371/journal.pone.0250925 (PMC8096087; doi:10.1371/journal.pone.0250925)

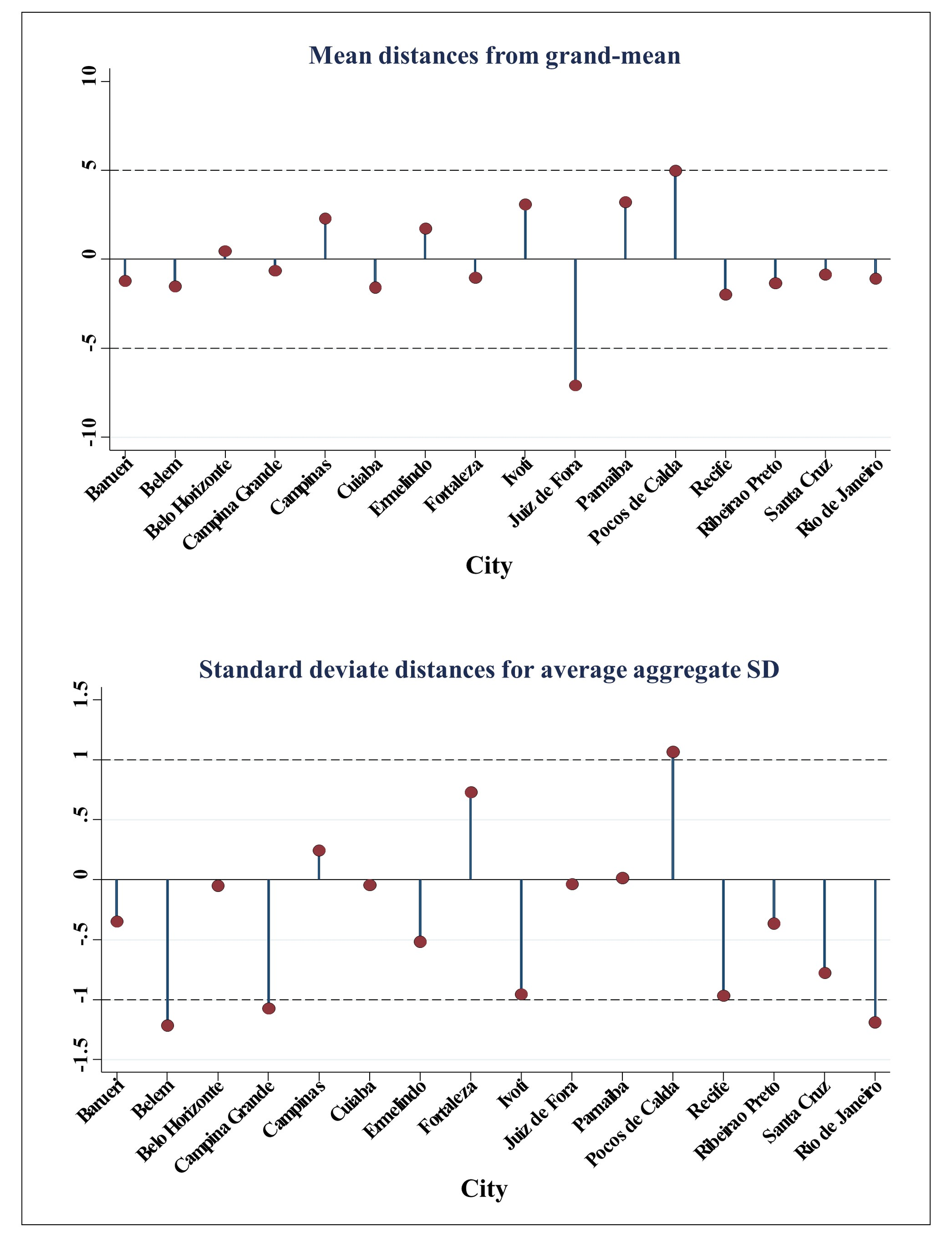

Supplement: S1 Fig — (TIF) [file pone.0250925.s002.tif]

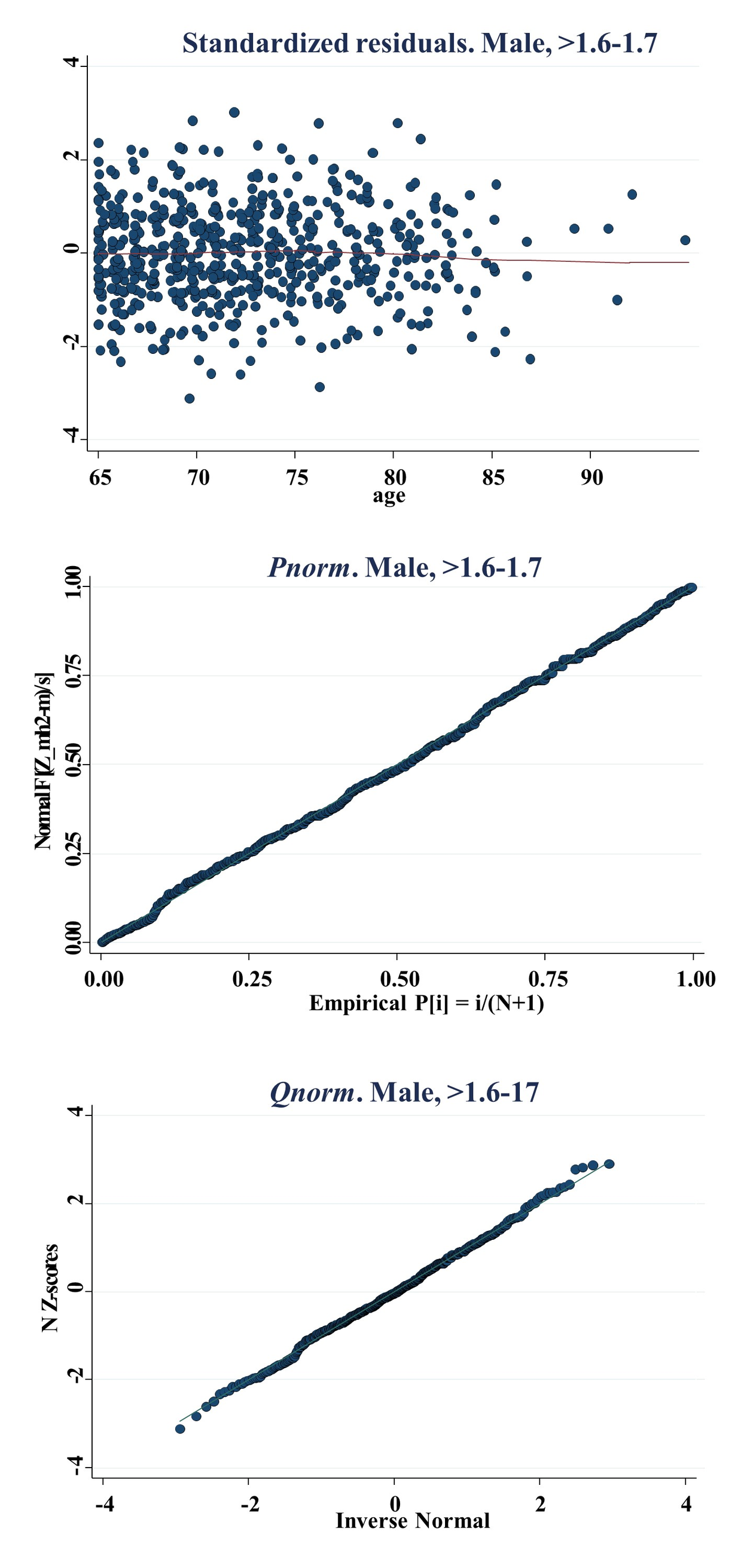

Supplement: S2 Fig — (TIF) [file pone.0250925.s003.tif]

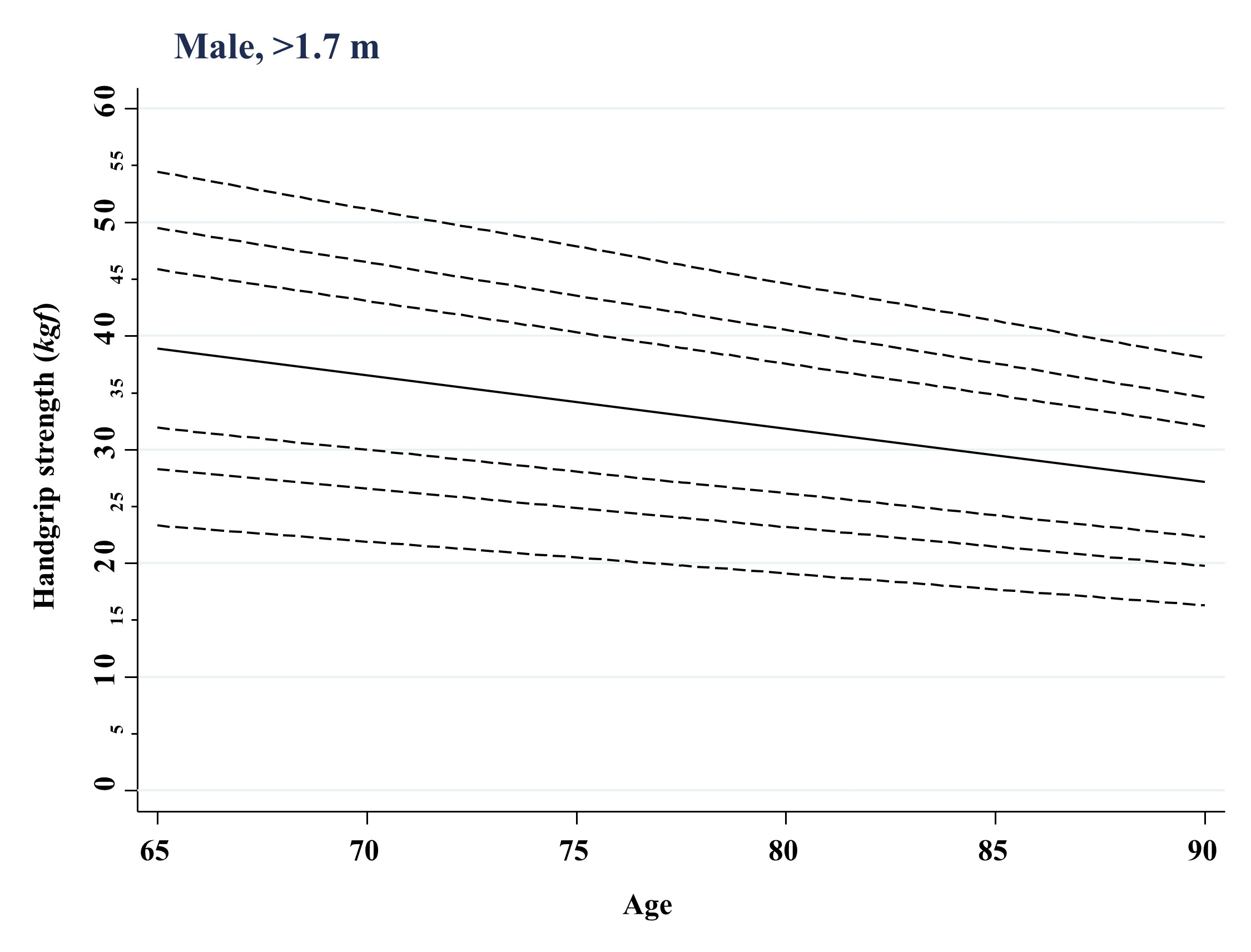

Supplement: S3 Fig — (TIF) [file pone.0250925.s004.tif]

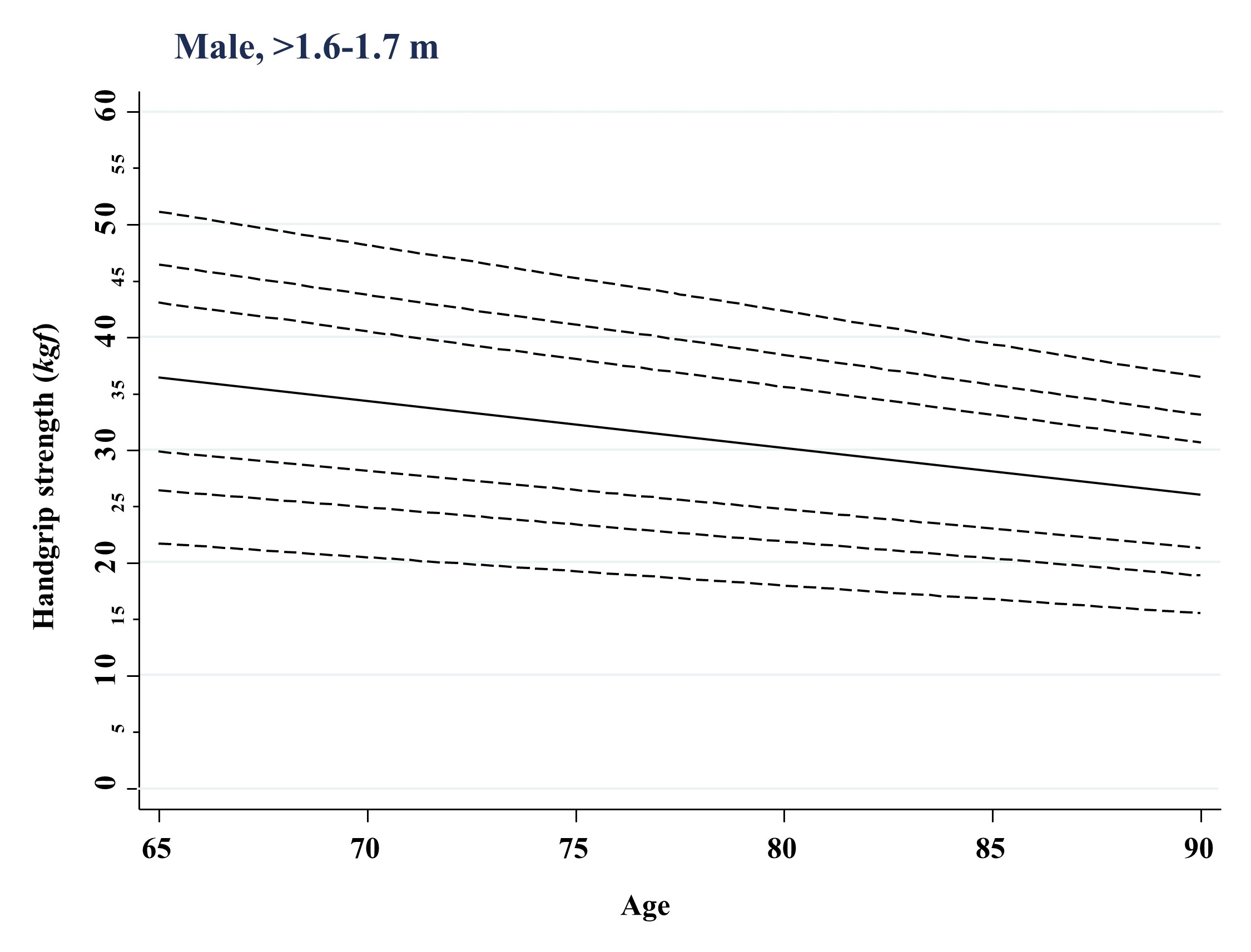

Supplement: S4 Fig — (TIF) [file pone.0250925.s005.tif]

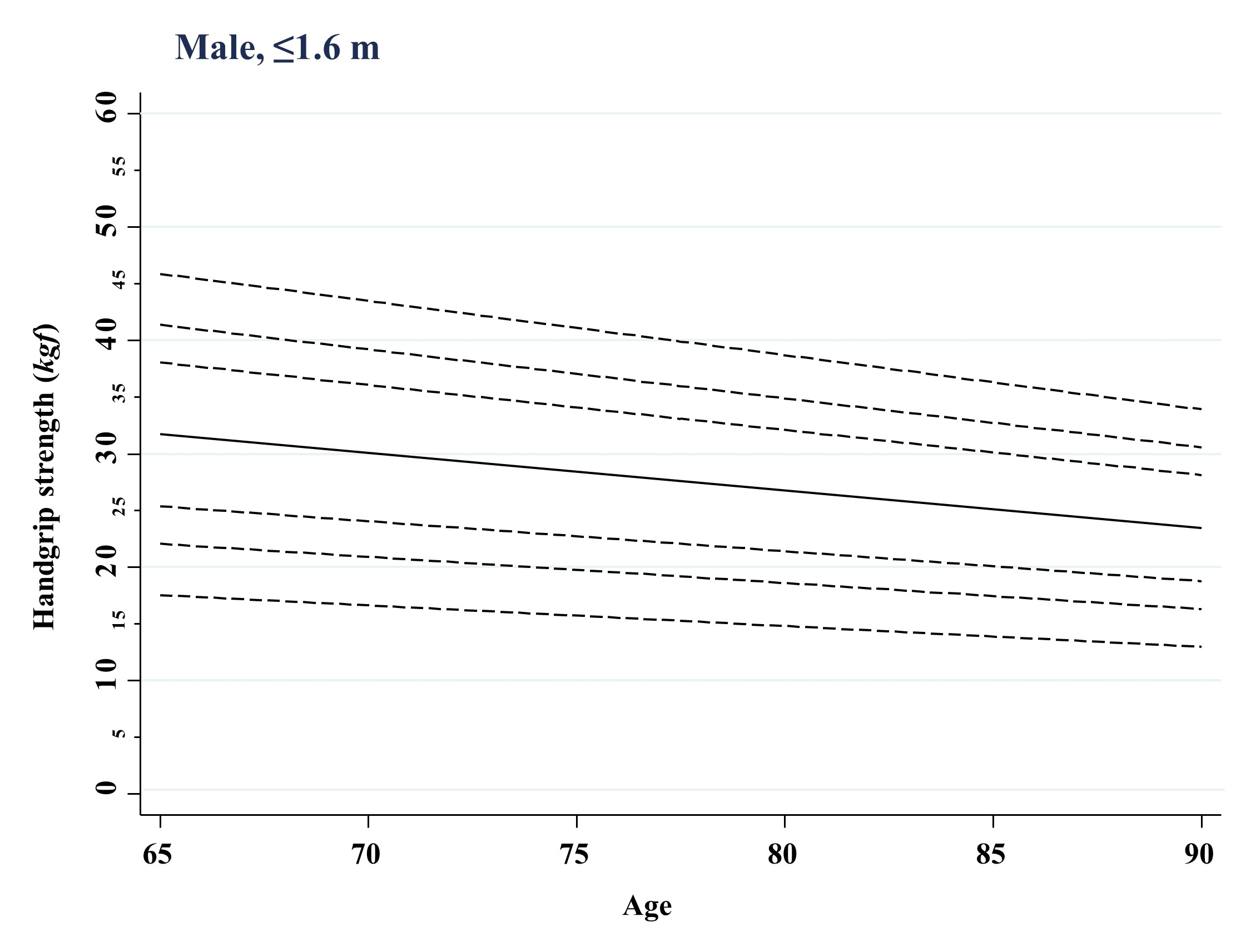

Supplement: S5 Fig — (TIF) [file pone.0250925.s006.tif]

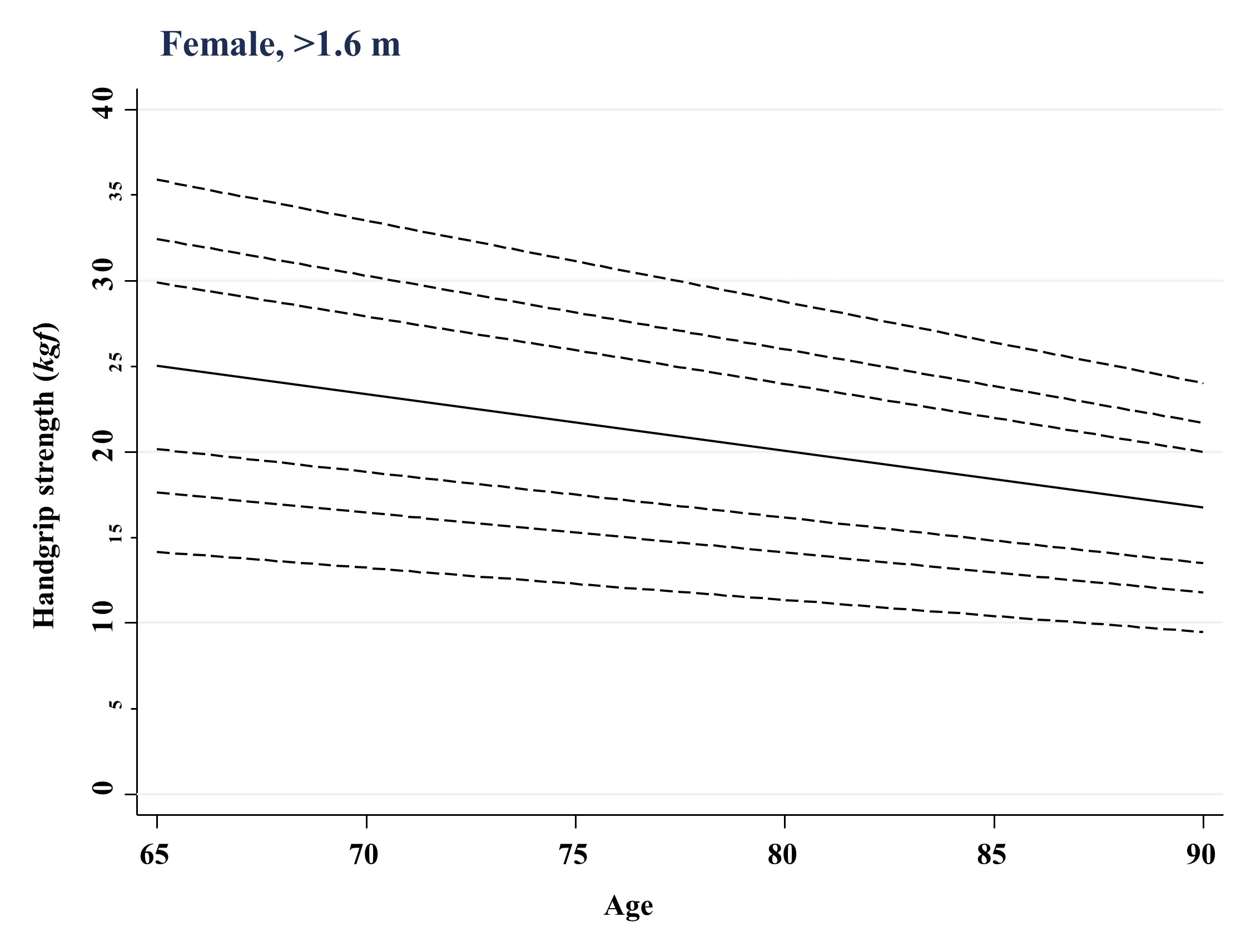

Supplement: S6 Fig — (TIF) [file pone.0250925.s007.tif]

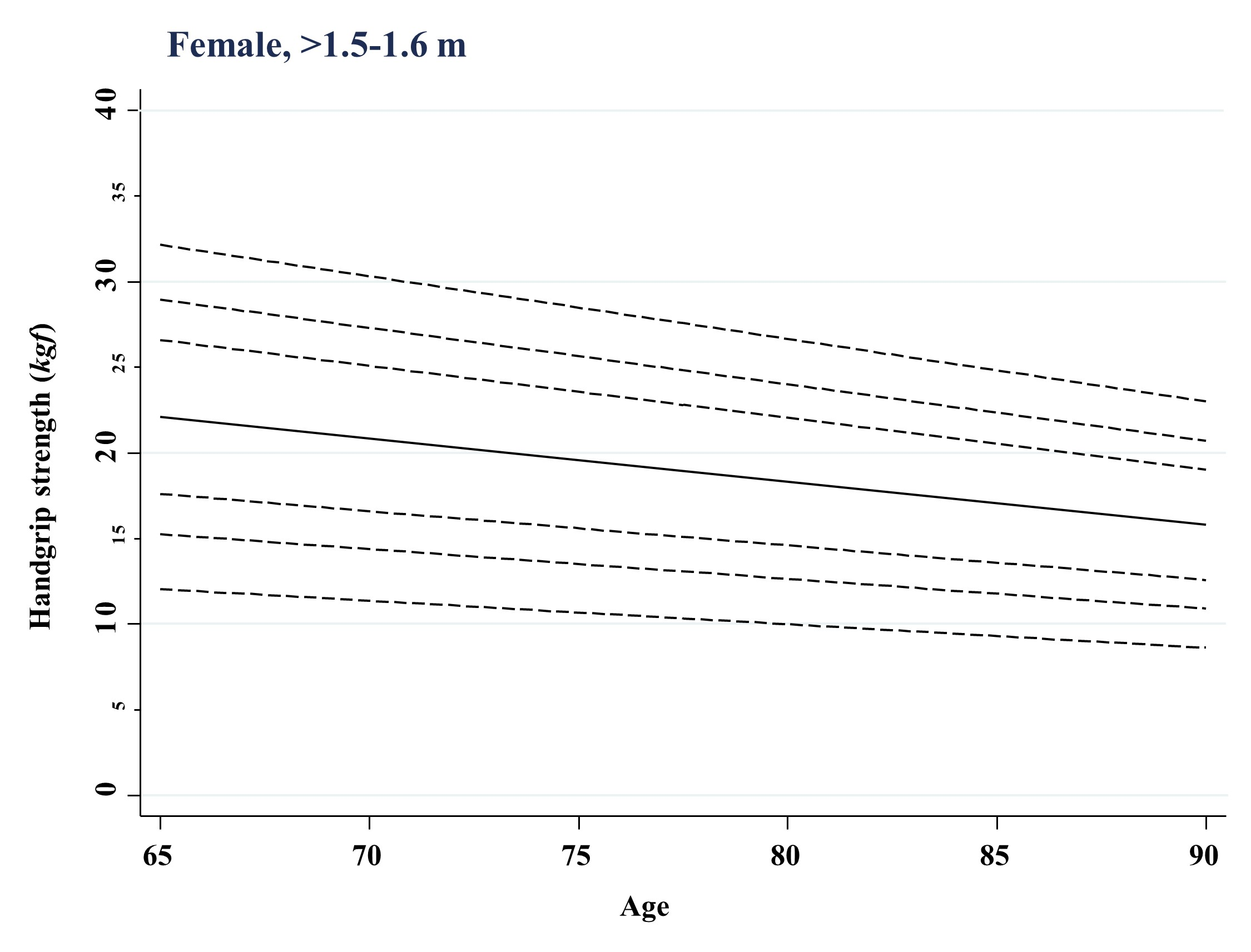

Supplement: S7 Fig — (TIF) [file pone.0250925.s008.tif]

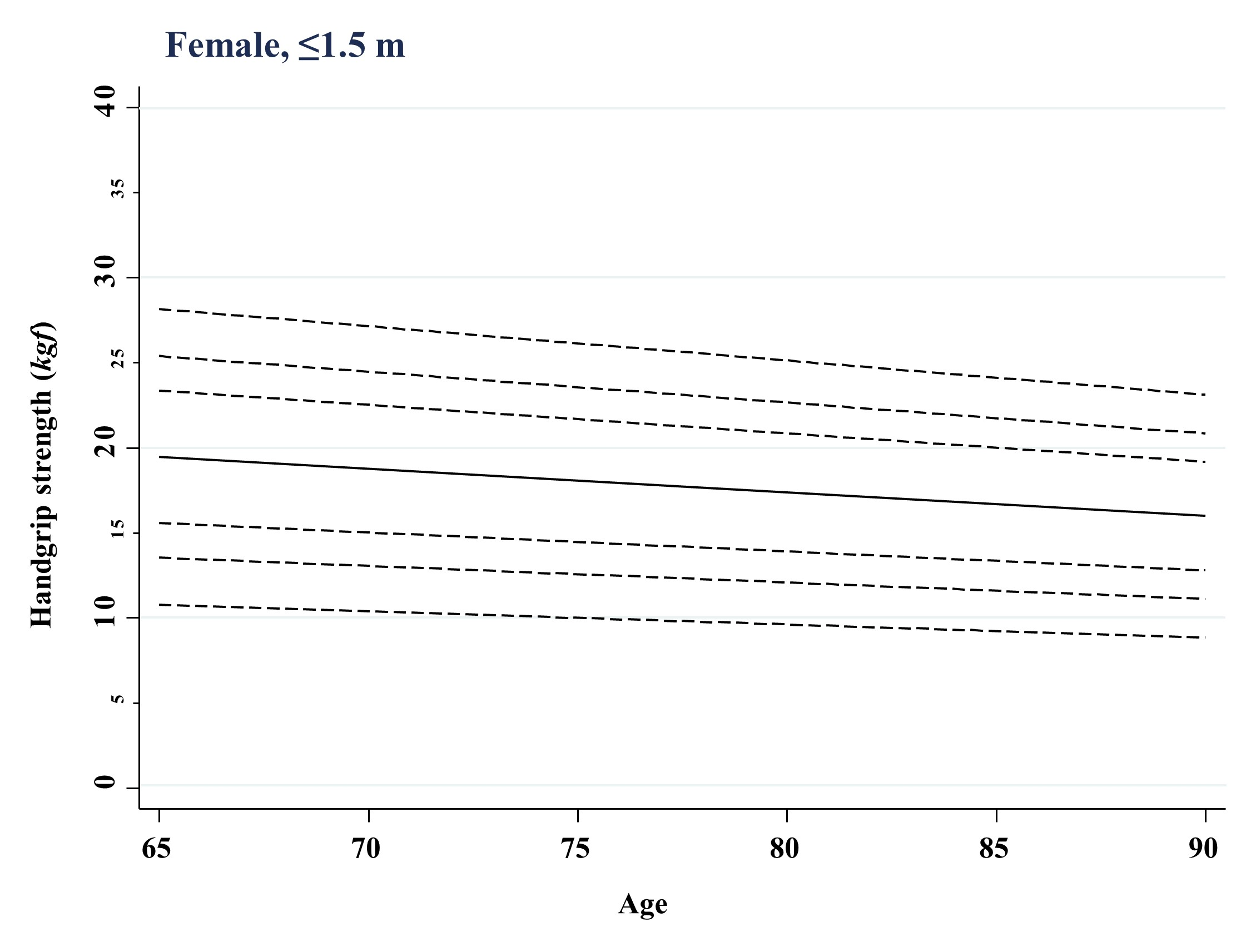

Supplement: S8 Fig — (TIF) [file pone.0250925.s009.tif]

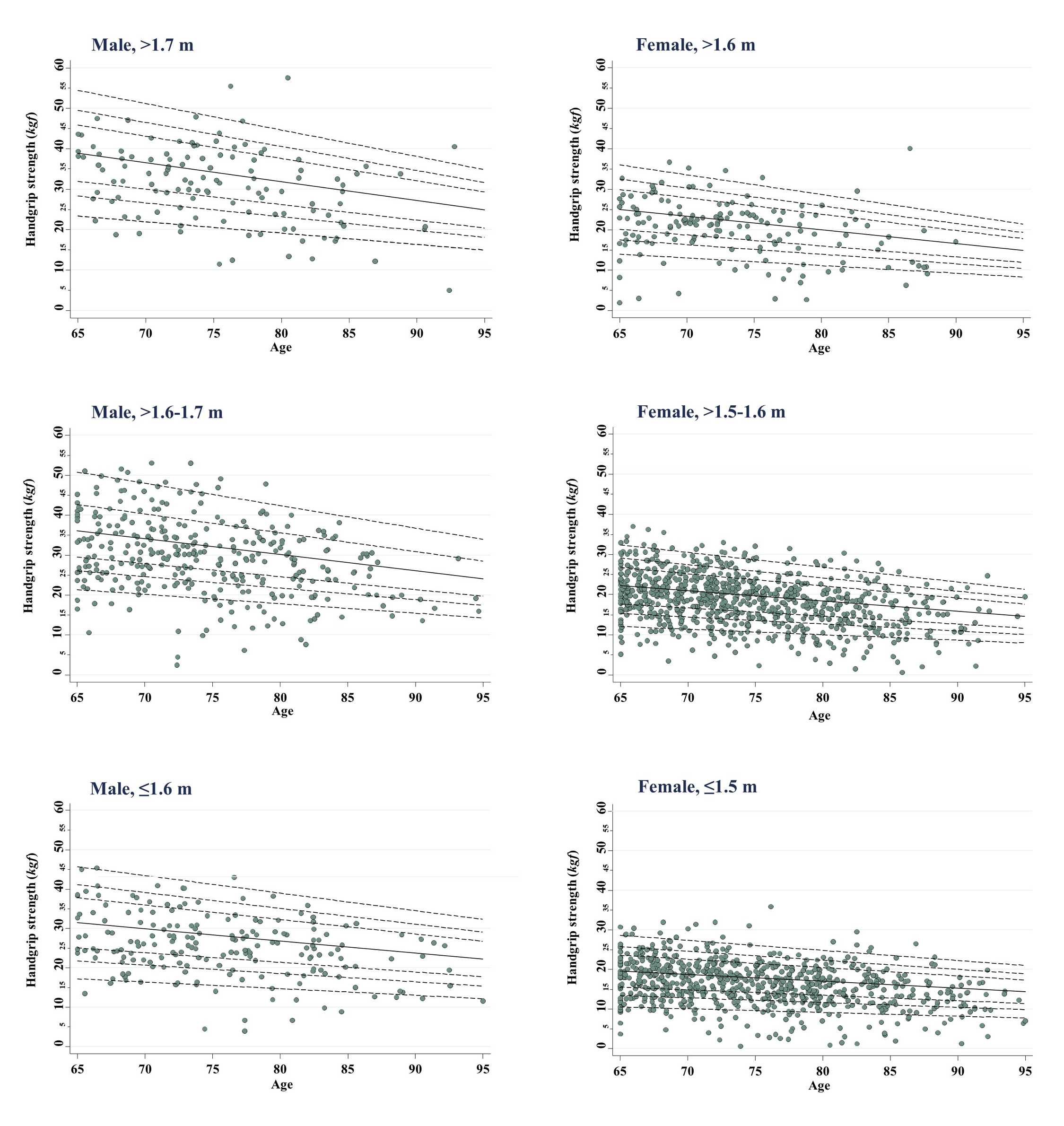

Supplement: S9 Fig — (TIF) [file pone.0250925.s010.tif]
