# Supplementary material for: Normative reference values of handgrip strength for Brazilian older people aged 65 to 90 years: Evidence from the multicenter Fibra‑BR study
Source: PLoS One. 2021 May 4;16(5):e0250925. doi: 10.1371/journal.pone.0250925 (PMC8096087; doi:10.1371/journal.pone.0250925)
Supplement: S1 Table — (DOCX) [file pone.0250925.s011.docx]

# **S1 Table. Hand grip strength (kgf) projected for male >1.7 meters for a wide array of centiles.**

| **Age** | **Centiles for HGS (*kgf*)** | | | | | | | | | | | | |
| --- | --- | --- | --- | --- | --- | --- | --- | --- | --- | --- | --- | --- | --- |
|  | **2.5** | **3** | **5** | **10** | **20** | **25** | **50** | **75** | **80** | **90** | **95** | **97** | **97.5** |
| 65 | 22.66 | 23.32 | 25.27 | 28.27 | 31.91 | 33.30 | 38.87 | 44.45 | 45.84 | 49.48 | 52.48 | 54.43 | 55.09 |
| 66 | 22.39 | 23.04 | 24.96 | 27.93 | 31.53 | 32.89 | 38.41 | 43.92 | 45.29 | 48.88 | 51.85 | 53.78 | 54.42 |
| 67 | 22.12 | 22.76 | 24.66 | 27.59 | 31.14 | 32.49 | 37.94 | 43.38 | 44.73 | 48.28 | 51.22 | 53.12 | 53.76 |
| 68 | 21.84 | 22.47 | 24.36 | 27.25 | 30.76 | 32.09 | 37.47 | 42.85 | 44.18 | 47.69 | 50.59 | 52.47 | 53.10 |
| 69 | 21.57 | 22.19 | 24.05 | 26.91 | 30.38 | 31.69 | 37.00 | 42.31 | 43.63 | 47.09 | 49.95 | 51.81 | 52.44 |
| 70 | 21.30 | 21.91 | 23.75 | 26.57 | 29.99 | 31.29 | 36.54 | 41.78 | 43.08 | 46.50 | 49.32 | 51.16 | 51.77 |
| 71 | 21.02 | 21.63 | 23.44 | 26.23 | 29.61 | 30.89 | 36.07 | 41.24 | 42.53 | 45.90 | 48.69 | 50.50 | 51.11 |
| 72 | 20.75 | 21.35 | 23.14 | 25.89 | 29.22 | 30.49 | 35.60 | 40.71 | 41.97 | 45.31 | 48.06 | 49.85 | 50.45 |
| 73 | 20.48 | 21.07 | 22.83 | 25.55 | 28.84 | 30.09 | 35.13 | 40.17 | 41.42 | 44.71 | 47.43 | 49.19 | 49.78 |
| 74 | 20.21 | 20.79 | 22.53 | 25.21 | 28.46 | 29.69 | 34.66 | 39.64 | 40.87 | 44.12 | 46.80 | 48.54 | 49.12 |
| 75 | 19.93 | 20.51 | 22.23 | 24.87 | 28.07 | 29.29 | 34.20 | 39.10 | 40.32 | 43.52 | 46.16 | 47.88 | 48.46 |
| 76 | 19.66 | 20.23 | 21.92 | 24.53 | 27.69 | 28.89 | 33.73 | 38.57 | 39.77 | 42.92 | 45.53 | 47.23 | 47.79 |
| 77 | 19.39 | 19.95 | 21.62 | 24.19 | 27.30 | 28.49 | 33.26 | 38.03 | 39.22 | 42.33 | 44.90 | 46.57 | 47.13 |
| 78 | 19.12 | 19.67 | 21.31 | 23.85 | 26.92 | 28.08 | 32.79 | 37.50 | 38.66 | 41.73 | 44.27 | 45.91 | 46.47 |
| 79 | 18.84 | 19.39 | 21.01 | 23.51 | 26.53 | 27.68 | 32.32 | 36.96 | 38.11 | 41.14 | 43.64 | 45.26 | 45.80 |
| 80 | 18.57 | 19.11 | 20.71 | 23.17 | 26.15 | 27.28 | 31.86 | 36.43 | 37.56 | 40.54 | 43.01 | 44.60 | 45.14 |
| 81 | 18.30 | 18.83 | 20.40 | 22.83 | 25.77 | 26.88 | 31.39 | 35.89 | 37.01 | 39.95 | 42.37 | 43.95 | 44.48 |
| 82 | 18.02 | 18.55 | 20.10 | 22.49 | 25.38 | 26.48 | 30.92 | 35.36 | 36.46 | 39.35 | 41.74 | 43.29 | 43.81 |
| 83 | 17.75 | 18.26 | 19.79 | 22.15 | 25.00 | 26.08 | 30.45 | 34.82 | 35.91 | 38.76 | 41.11 | 42.64 | 43.15 |
| 84 | 17.48 | 17.98 | 19.49 | 21.81 | 24.61 | 25.68 | 29.98 | 34.29 | 35.35 | 38.16 | 40.48 | 41.98 | 42.49 |
| 85 | 17.21 | 17.70 | 19.18 | 21.47 | 24.23 | 25.28 | 29.52 | 33.75 | 34.80 | 37.56 | 39.85 | 41.33 | 41.83 |
| 86 | 16.93 | 17.42 | 18.88 | 21.13 | 23.85 | 24.88 | 29.05 | 33.22 | 34.25 | 36.97 | 39.21 | 40.67 | 41.16 |
| 87 | 16.66 | 17.14 | 18.58 | 20.79 | 23.46 | 24.48 | 28.58 | 32.68 | 33.70 | 36.37 | 38.58 | 40.02 | 40.50 |
| 88 | 16.39 | 16.86 | 18.27 | 20.45 | 23.08 | 24.08 | 28.11 | 32.15 | 33.15 | 35.78 | 37.95 | 39.36 | 39.84 |
| 89 | 16.11 | 16.58 | 17.97 | 20.11 | 22.69 | 23.68 | 27.64 | 31.61 | 32.59 | 35.18 | 37.32 | 38.71 | 39.17 |
| 90 | 15.84 | 16.30 | 17.66 | 19.76 | 22.31 | 23.28 | 27.18 | 31.08 | 32.04 | 34.59 | 36.69 | 38.05 | 38.51 |
| 91 | 15.57 | 16.02 | 17.36 | 19.42 | 21.92 | 22.87 | 26.71 | 30.54 | 31.49 | 33.99 | 36.06 | 37.40 | 37.85 |
| 92 | 15.30 | 15.74 | 17.06 | 19.08 | 21.54 | 22.47 | 26.24 | 30.01 | 30.94 | 33.40 | 35.42 | 36.74 | 37.18 |
| 93 | 15.02 | 15.46 | 16.75 | 18.74 | 21.16 | 22.07 | 25.77 | 29.47 | 30.39 | 32.80 | 34.79 | 36.09 | 36.52 |
| 94 | 14.75 | 15.18 | 16.45 | 18.40 | 20.77 | 21.67 | 25.30 | 28.94 | 29.84 | 32.20 | 34.16 | 35.43 | 35.86 |
| 95 | 14.48 | 14.90 | 16.14 | 18.06 | 20.39 | 21.27 | 24.84 | 28.40 | 29.28 | 31.61 | 33.53 | 34.78 | 35.19 |
