# Supplementary material for: Normative reference values of handgrip strength for Brazilian older people aged 65 to 90 years: Evidence from the multicenter Fibra‑BR study
Source: PLoS One. 2021 May 4;16(5):e0250925. doi: 10.1371/journal.pone.0250925 (PMC8096087; doi:10.1371/journal.pone.0250925)
Supplement: S2 Table — (DOCX) [file pone.0250925.s012.docx]

# **S2 Table. Hand grip strength (*kgf*) projected for male from >1.6 to 1.7 meters for a wide array of centiles.**

| **Age** | **Centiles for HGS (*kgf*)** | | | | | | | | | | | | |
| --- | --- | --- | --- | --- | --- | --- | --- | --- | --- | --- | --- | --- | --- |
|  | **2.5** | **3** | **5** | **10** | **20** | **25** | **50** | **75** | **80** | **90** | **95** | **97** | **97.5** |
| 65 | 35.98 | 36.00 | 36.05 | 36.13 | 36.22 | 36.26 | 36.40 | 36.55 | 36.59 | 36.68 | 36.76 | 36.81 | 36.83 |
| 66 | 35.57 | 35.58 | 35.63 | 35.71 | 35.81 | 35.84 | 35.99 | 36.13 | 36.17 | 36.26 | 36.34 | 36.39 | 36.41 |
| 67 | 35.15 | 35.17 | 35.22 | 35.29 | 35.39 | 35.42 | 35.57 | 35.71 | 35.75 | 35.85 | 35.92 | 35.97 | 35.99 |
| 68 | 34.73 | 34.75 | 34.80 | 34.88 | 34.97 | 35.01 | 35.15 | 35.30 | 35.33 | 35.43 | 35.51 | 35.56 | 35.57 |
| 69 | 34.31 | 34.33 | 34.38 | 34.46 | 34.55 | 34.59 | 34.74 | 34.88 | 34.92 | 35.01 | 35.09 | 35.14 | 35.16 |
| 70 | 33.90 | 33.91 | 33.96 | 34.04 | 34.14 | 34.17 | 34.32 | 34.46 | 34.50 | 34.59 | 34.67 | 34.72 | 34.74 |
| 71 | 33.48 | 33.50 | 33.55 | 33.63 | 33.72 | 33.76 | 33.90 | 34.05 | 34.08 | 34.18 | 34.26 | 34.31 | 34.32 |
| 72 | 33.06 | 33.08 | 33.13 | 33.21 | 33.30 | 33.34 | 33.48 | 33.63 | 33.67 | 33.76 | 33.84 | 33.89 | 33.91 |
| 73 | 32.65 | 32.66 | 32.71 | 32.79 | 32.89 | 32.92 | 33.07 | 33.21 | 33.25 | 33.34 | 33.42 | 33.47 | 33.49 |
| 74 | 32.23 | 32.25 | 32.30 | 32.37 | 32.47 | 32.50 | 32.65 | 32.79 | 32.83 | 32.93 | 33.00 | 33.05 | 33.07 |
| 75 | 31.81 | 31.83 | 31.88 | 31.96 | 32.05 | 32.09 | 32.23 | 32.38 | 32.41 | 32.51 | 32.59 | 32.64 | 32.65 |
| 76 | 31.39 | 31.41 | 31.46 | 31.54 | 31.63 | 31.67 | 31.82 | 31.96 | 32.00 | 32.09 | 32.17 | 32.22 | 32.24 |
| 77 | 30.98 | 30.99 | 31.04 | 31.12 | 31.22 | 31.25 | 31.40 | 31.54 | 31.58 | 31.67 | 31.75 | 31.80 | 31.82 |
| 78 | 30.56 | 30.58 | 30.63 | 30.71 | 30.80 | 30.84 | 30.98 | 31.13 | 31.16 | 31.26 | 31.33 | 31.39 | 31.40 |
| 79 | 30.14 | 30.16 | 30.21 | 30.29 | 30.38 | 30.42 | 30.56 | 30.71 | 30.75 | 30.84 | 30.92 | 30.97 | 30.99 |
| 80 | 29.73 | 29.74 | 29.79 | 29.87 | 29.97 | 30.00 | 30.15 | 30.29 | 30.33 | 30.42 | 30.50 | 30.55 | 30.57 |
| 81 | 29.31 | 29.33 | 29.38 | 29.45 | 29.55 | 29.58 | 29.73 | 29.87 | 29.91 | 30.01 | 30.08 | 30.13 | 30.15 |
| 82 | 28.89 | 28.91 | 28.96 | 29.04 | 29.13 | 29.17 | 29.31 | 29.46 | 29.49 | 29.59 | 29.67 | 29.72 | 29.73 |
| 83 | 28.47 | 28.49 | 28.54 | 28.62 | 28.71 | 28.75 | 28.90 | 29.04 | 29.08 | 29.17 | 29.25 | 29.30 | 29.32 |
| 84 | 28.06 | 28.07 | 28.12 | 28.20 | 28.30 | 28.33 | 28.48 | 28.62 | 28.66 | 28.75 | 28.83 | 28.88 | 28.90 |
| 85 | 27.64 | 27.66 | 27.71 | 27.79 | 27.88 | 27.92 | 28.06 | 28.21 | 28.24 | 28.34 | 28.41 | 28.47 | 28.48 |
| 86 | 27.22 | 27.24 | 27.29 | 27.37 | 27.46 | 27.50 | 28.06 | 27.79 | 27.82 | 27.92 | 28.00 | 28.05 | 28.07 |
| 87 | 26.81 | 26.82 | 26.87 | 26.95 | 27.05 | 27.08 | 28.06 | 27.37 | 27.41 | 27.50 | 27.58 | 27.63 | 27.65 |
| 88 | 26.39 | 26.41 | 26.46 | 26.53 | 26.63 | 26.66 | 28.06 | 26.95 | 26.99 | 27.09 | 27.16 | 27.21 | 27.23 |
| 89 | 25.97 | 25.99 | 26.04 | 26.12 | 26.21 | 26.25 | 28.06 | 26.54 | 26.57 | 26.67 | 26.75 | 26.80 | 26.81 |
| 90 | 25.55 | 25.57 | 25.62 | 25.70 | 25.79 | 25.83 | 28.06 | 26.12 | 26.16 | 26.25 | 26.33 | 26.38 | 26.40 |
| 91 | 25.14 | 25.15 | 25.20 | 25.28 | 25.38 | 25.41 | 28.06 | 25.70 | 25.74 | 25.83 | 25.91 | 25.96 | 25.98 |
| 92 | 24.72 | 24.74 | 24.79 | 24.87 | 24.96 | 25.00 | 28.06 | 25.29 | 25.32 | 25.42 | 25.49 | 25.55 | 25.56 |
| 93 | 24.30 | 24.32 | 24.37 | 24.45 | 24.54 | 24.58 | 28.06 | 24.87 | 24.90 | 25.00 | 25.08 | 25.13 | 25.15 |
| 94 | 23.89 | 23.90 | 23.95 | 24.03 | 24.13 | 24.16 | 28.06 | 24.45 | 24.49 | 24.58 | 24.66 | 24.71 | 24.73 |
| 95 | 23.47 | 23.49 | 23.54 | 23.61 | 23.71 | 23.74 | 28.06 | 24.03 | 24.07 | 24.17 | 24.24 | 24.29 | 24.31 |
