# Supplementary material for: Normative reference values of handgrip strength for Brazilian older people aged 65 to 90 years: Evidence from the multicenter Fibra‑BR study
Source: PLoS One. 2021 May 4;16(5):e0250925. doi: 10.1371/journal.pone.0250925 (PMC8096087; doi:10.1371/journal.pone.0250925)
Supplement: S3 Table — (DOCX) [file pone.0250925.s013.docx]

# **S3 Table. Hand grip strength (*kgf*) projected for male ≤1.6 meters for a wide array of centiles.**

| **Age** | **Centiles for HGS (kgf)** | | | | | | | | | | | | |
| --- | --- | --- | --- | --- | --- | --- | --- | --- | --- | --- | --- | --- | --- |
|  | **2.5** | **3** | **5** | **10** | **20** | **25** | **50** | **75** | **80** | **90** | **95** | **97** | **97.5** |
| 65 | 16.95 | 17.55 | 19.32 | 22.06 | 25.37 | 26.63 | 31.71 | 36.79 | 38.04 | 41.36 | 44.09 | 45.87 | 46.47 |
| 66 | 16.77 | 17.36 | 19.12 | 21.83 | 25.11 | 26.35 | 31.38 | 36.40 | 37.65 | 40.93 | 43.63 | 45.39 | 45.98 |
| 67 | 16.60 | 17.18 | 18.92 | 21.60 | 24.84 | 26.07 | 31.05 | 36.02 | 37.25 | 40.49 | 43.17 | 44.91 | 45.50 |
| 68 | 16.42 | 17.00 | 18.72 | 21.37 | 24.58 | 25.80 | 30.71 | 35.63 | 36.85 | 40.06 | 42.71 | 44.43 | 45.01 |
| 69 | 16.24 | 16.81 | 18.52 | 21.14 | 24.31 | 25.52 | 30.38 | 35.25 | 36.46 | 39.63 | 42.25 | 43.95 | 44.53 |
| 70 | 16.07 | 16.63 | 18.31 | 20.91 | 24.05 | 25.24 | 30.05 | 34.87 | 36.06 | 39.20 | 41.79 | 43.48 | 44.04 |
| 71 | 15.89 | 16.45 | 18.11 | 20.68 | 23.78 | 24.96 | 29.72 | 34.48 | 35.66 | 38.77 | 41.33 | 43.00 | 43.56 |
| 72 | 15.71 | 16.26 | 17.91 | 20.45 | 23.52 | 24.68 | 29.39 | 34.10 | 35.27 | 38.34 | 40.87 | 42.52 | 43.07 |
| 73 | 15.53 | 16.08 | 17.71 | 20.22 | 23.25 | 24.41 | 29.06 | 33.72 | 34.87 | 37.91 | 40.41 | 42.04 | 42.59 |
| 74 | 15.36 | 15.90 | 17.51 | 19.99 | 22.99 | 24.13 | 28.73 | 33.33 | 34.47 | 37.47 | 39.95 | 41.56 | 42.10 |
| 75 | 15.18 | 15.71 | 17.31 | 19.76 | 22.72 | 23.85 | 28.40 | 32.95 | 34.08 | 37.04 | 39.49 | 41.08 | 41.62 |
| 76 | 15.00 | 15.53 | 17.10 | 19.53 | 22.46 | 23.57 | 28.07 | 32.56 | 33.68 | 36.61 | 39.03 | 40.60 | 41.13 |
| 77 | 14.83 | 15.35 | 16.90 | 19.30 | 22.19 | 23.29 | 27.74 | 32.18 | 33.28 | 36.18 | 38.57 | 40.13 | 40.65 |
| 78 | 14.65 | 15.17 | 16.70 | 19.07 | 21.93 | 23.02 | 27.41 | 31.80 | 32.88 | 35.75 | 38.11 | 39.65 | 40.16 |
| 79 | 14.47 | 14.98 | 16.50 | 18.84 | 21.66 | 22.74 | 27.08 | 31.41 | 32.49 | 35.32 | 37.65 | 39.17 | 39.68 |
| 80 | 14.30 | 14.80 | 16.30 | 18.61 | 21.40 | 22.46 | 26.75 | 31.03 | 32.09 | 34.88 | 37.19 | 38.69 | 39.19 |
| 81 | 14.12 | 14.62 | 16.10 | 18.38 | 21.14 | 22.18 | 26.41 | 30.65 | 31.69 | 34.45 | 36.73 | 38.21 | 38.71 |
| 82 | 13.94 | 14.43 | 15.90 | 18.15 | 20.87 | 21.91 | 26.08 | 30.26 | 31.30 | 34.02 | 36.27 | 37.73 | 38.22 |
| 83 | 13.77 | 14.25 | 15.69 | 17.92 | 20.61 | 21.63 | 25.75 | 29.88 | 30.90 | 33.59 | 35.81 | 37.26 | 37.74 |
| 84 | 13.59 | 14.07 | 15.49 | 17.69 | 20.34 | 21.35 | 25.42 | 29.49 | 30.50 | 33.16 | 35.35 | 36.78 | 37.25 |
| 85 | 13.41 | 13.88 | 15.29 | 17.46 | 20.08 | 21.07 | 25.09 | 29.11 | 30.11 | 32.73 | 34.89 | 36.30 | 36.77 |
| 86 | 13.24 | 13.70 | 15.09 | 17.22 | 19.81 | 20.79 | 24.76 | 28.73 | 29.71 | 32.30 | 34.43 | 35.82 | 36.29 |
| 87 | 13.06 | 13.52 | 14.89 | 16.99 | 19.55 | 20.52 | 24.43 | 28.34 | 29.31 | 31.86 | 33.97 | 35.34 | 35.80 |
| 88 | 12.88 | 13.34 | 14.69 | 16.76 | 19.28 | 20.24 | 24.10 | 27.96 | 28.92 | 31.43 | 33.51 | 34.86 | 35.32 |
| 89 | 12.71 | 13.15 | 14.48 | 16.53 | 19.02 | 19.96 | 23.77 | 27.58 | 28.52 | 31.00 | 33.05 | 34.38 | 34.83 |
| 90 | 12.53 | 12.97 | 14.28 | 16.30 | 18.75 | 19.68 | 23.44 | 27.19 | 28.12 | 30.57 | 32.59 | 33.91 | 34.35 |
| 91 | 12.35 | 12.79 | 14.08 | 16.07 | 18.49 | 19.41 | 23.11 | 26.81 | 27.72 | 30.14 | 32.13 | 33.43 | 33.86 |
| 92 | 12.17 | 12.60 | 13.88 | 15.84 | 18.22 | 19.13 | 22.78 | 26.42 | 27.33 | 29.71 | 31.67 | 32.95 | 33.38 |
| 93 | 12.00 | 12.42 | 13.68 | 15.61 | 17.96 | 18.85 | 22.44 | 26.04 | 26.93 | 29.28 | 31.21 | 32.47 | 32.89 |
| 94 | 11.82 | 12.24 | 13.48 | 15.38 | 17.69 | 18.57 | 22.11 | 25.66 | 26.53 | 28.84 | 30.75 | 31.99 | 32.41 |
| 95 | 11.64 | 12.05 | 13.27 | 15.15 | 17.43 | 18.29 | 21.78 | 25.27 | 26.14 | 28.41 | 30.29 | 31.51 | 31.92 |
