# Supplementary material for: Normative reference values of handgrip strength for Brazilian older people aged 65 to 90 years: Evidence from the multicenter Fibra‑BR study
Source: PLoS One. 2021 May 4;16(5):e0250925. doi: 10.1371/journal.pone.0250925 (PMC8096087; doi:10.1371/journal.pone.0250925)
Supplement: S4 Table — (DOCX) [file pone.0250925.s014.docx]

# **S4 Table. Hand grip strength (*kgf*) projected for female >1.6 meters for a wide array of centiles.**

| **Age** | **Centiles for HGS (kgf)** | | | | | | | | | | | | |
| --- | --- | --- | --- | --- | --- | --- | --- | --- | --- | --- | --- | --- | --- |
|  | **2.5** | **3** | **5** | **10** | **20** | **25** | **50** | **75** | **80** | **90** | **95** | **97** | **97.5** |
| 65 | 13.70 | 14.15 | 15.52 | 17.62 | 20.16 | 21.12 | 25.02 | 28.92 | 29.88 | 32.42 | 34.52 | 35.89 | 36.34 |
| 66 | 13.52 | 13.97 | 15.31 | 17.38 | 19.89 | 20.84 | 24.69 | 28.53 | 29.49 | 31.99 | 34.07 | 35.41 | 35.86 |
| 67 | 13.33 | 13.78 | 15.11 | 17.15 | 19.62 | 20.56 | 24.36 | 28.15 | 29.09 | 31.56 | 33.61 | 34.94 | 35.38 |
| 68 | 13.15 | 13.59 | 14.90 | 16.92 | 19.36 | 20.28 | 24.03 | 27.77 | 28.70 | 31.14 | 33.15 | 34.46 | 34.90 |
| 69 | 12.97 | 13.40 | 14.70 | 16.68 | 19.09 | 20.00 | 23.69 | 27.39 | 28.30 | 30.71 | 32.69 | 33.98 | 34.42 |
| 70 | 12.79 | 13.22 | 14.49 | 16.45 | 18.82 | 19.72 | 23.36 | 27.00 | 27.90 | 30.28 | 32.24 | 33.51 | 33.94 |
| 71 | 12.61 | 13.03 | 14.28 | 16.22 | 18.56 | 19.45 | 23.03 | 26.62 | 27.51 | 29.85 | 31.78 | 33.03 | 33.46 |
| 72 | 12.43 | 12.84 | 14.08 | 15.98 | 18.29 | 19.17 | 22.70 | 26.24 | 27.11 | 29.42 | 31.32 | 32.56 | 32.97 |
| 73 | 12.25 | 12.65 | 13.87 | 15.75 | 18.02 | 18.89 | 22.37 | 25.85 | 26.72 | 28.99 | 30.87 | 32.08 | 32.49 |
| 74 | 12.06 | 12.47 | 13.67 | 15.52 | 17.76 | 18.61 | 22.04 | 25.47 | 26.32 | 28.56 | 30.41 | 31.61 | 32.01 |
| 75 | 11.88 | 12.28 | 13.46 | 15.28 | 17.49 | 18.33 | 21.71 | 25.09 | 25.93 | 28.13 | 29.95 | 31.13 | 31.53 |
| 76 | 11.70 | 12.09 | 13.26 | 15.05 | 17.22 | 18.05 | 21.38 | 24.70 | 25.53 | 27.70 | 29.49 | 30.66 | 31.05 |
| 77 | 11.52 | 11.91 | 13.05 | 14.82 | 16.95 | 17.77 | 21.04 | 24.32 | 25.13 | 27.27 | 29.04 | 30.18 | 30.57 |
| 78 | 11.34 | 11.72 | 12.85 | 14.58 | 16.69 | 17.49 | 20.71 | 23.94 | 24.74 | 26.84 | 28.58 | 29.71 | 30.09 |
| 79 | 11.16 | 11.53 | 12.64 | 14.35 | 16.42 | 17.21 | 20.38 | 23.56 | 24.34 | 26.41 | 28.12 | 29.23 | 29.61 |
| 80 | 10.98 | 11.34 | 12.44 | 14.12 | 16.15 | 16.93 | 20.05 | 23.17 | 23.95 | 25.98 | 27.67 | 28.76 | 29.12 |
| 81 | 10.80 | 11.16 | 12.23 | 13.88 | 15.89 | 16.65 | 19.72 | 22.79 | 23.55 | 25.55 | 27.21 | 28.28 | 28.64 |
| 82 | 10.61 | 10.97 | 12.02 | 13.65 | 15.62 | 16.37 | 19.39 | 22.41 | 23.16 | 25.13 | 26.75 | 27.81 | 28.16 |
| 83 | 10.43 | 10.78 | 11.82 | 13.42 | 15.35 | 16.09 | 19.06 | 22.02 | 22.76 | 24.70 | 26.29 | 27.33 | 27.68 |
| 84 | 10.25 | 10.59 | 11.61 | 13.18 | 15.09 | 15.81 | 18.73 | 21.64 | 22.36 | 24.27 | 25.84 | 26.86 | 27.20 |
| 85 | 10.07 | 10.41 | 11.41 | 12.95 | 14.82 | 15.53 | 18.39 | 21.26 | 21.97 | 23.84 | 25.38 | 26.38 | 26.72 |
| 86 | 9.89 | 10.22 | 11.20 | 12.72 | 14.55 | 15.25 | 18.06 | 20.88 | 21.57 | 23.41 | 24.92 | 25.91 | 26.24 |
| 87 | 9.71 | 10.03 | 11.00 | 12.48 | 14.29 | 14.97 | 17.73 | 20.49 | 21.18 | 22.98 | 24.47 | 25.43 | 25.76 |
| 88 | 9.53 | 9.84 | 10.79 | 12.25 | 14.02 | 14.69 | 17.40 | 20.11 | 20.78 | 22.55 | 24.01 | 24.96 | 25.28 |
| 89 | 9.34 | 9.66 | 10.59 | 12.02 | 13.75 | 14.41 | 17.07 | 19.73 | 20.39 | 22.12 | 23.55 | 24.48 | 24.79 |
| 90 | 9.16 | 9.47 | 10.38 | 11.79 | 13.49 | 14.13 | 16.74 | 19.34 | 19.99 | 21.69 | 23.10 | 24.01 | 24.31 |
| 91 | 8.98 | 9.28 | 10.18 | 11.55 | 13.22 | 13.85 | 16.41 | 18.96 | 19.60 | 21.26 | 22.64 | 23.53 | 23.83 |
| 92 | 8.80 | 9.09 | 9.97 | 11.32 | 12.95 | 13.57 | 16.08 | 18.58 | 19.20 | 20.83 | 22.18 | 23.06 | 23.35 |
| 93 | 8.62 | 8.91 | 9.76 | 11.09 | 12.68 | 13.29 | 15.74 | 18.20 | 18.80 | 20.40 | 21.72 | 22.58 | 22.87 |
| 94 | 8.44 | 8.72 | 9.56 | 10.85 | 12.42 | 13.01 | 15.41 | 17.81 | 18.41 | 19.97 | 21.27 | 22.11 | 22.39 |
| 95 | 8.26 | 8.53 | 9.35 | 10.62 | 12.15 | 12.73 | 15.08 | 17.43 | 18.01 | 19.54 | 20.81 | 21.63 | 21.91 |
