# Supplementary material for: Normative reference values of handgrip strength for Brazilian older people aged 65 to 90 years: Evidence from the multicenter Fibra‑BR study
Source: PLoS One. 2021 May 4;16(5):e0250925. doi: 10.1371/journal.pone.0250925 (PMC8096087; doi:10.1371/journal.pone.0250925)
Supplement: S5 Table — (DOCX) [file pone.0250925.s015.docx]

# **S5 Table. Hand grip strength (*kgf*) projected for female from >1.5 to 1.6 meters for a wide array of centiles.**

| **Age** | **Centiles for HGS (kgf)** | | | | | | | | | | | | |
| --- | --- | --- | --- | --- | --- | --- | --- | --- | --- | --- | --- | --- | --- |
|  | **2.5** | **3** | **5** | **10** | **20** | **25** | **50** | **75** | **80** | **90** | **95** | **97** | **97.5** |
| 65 | 11.62 | 12.04 | 13.30 | 15.24 | 17.60 | 18.49 | 22.09 | 25.70 | 26.59 | 28.94 | 30.88 | 32.14 | 32.56 |
| 66 | 11.49 | 11.91 | 13.15 | 15.07 | 17.39 | 18.28 | 21.84 | 25.40 | 26.29 | 28.61 | 30.53 | 31.77 | 32.19 |
| 67 | 11.36 | 11.77 | 13.00 | 14.90 | 17.19 | 18.07 | 21.59 | 25.11 | 25.98 | 28.28 | 30.18 | 31.41 | 31.82 |
| 68 | 11.22 | 11.63 | 12.85 | 14.72 | 16.99 | 17.86 | 21.34 | 24.82 | 25.68 | 27.95 | 29.82 | 31.04 | 31.45 |
| 69 | 11.09 | 11.49 | 12.70 | 14.55 | 16.79 | 17.65 | 21.08 | 24.52 | 25.38 | 27.62 | 29.47 | 30.68 | 31.08 |
| 70 | 10.96 | 11.36 | 12.55 | 14.38 | 16.59 | 17.43 | 20.83 | 24.23 | 25.07 | 27.29 | 29.12 | 30.31 | 30.71 |
| 71 | 10.83 | 11.22 | 12.39 | 14.20 | 16.39 | 17.22 | 20.58 | 23.94 | 24.77 | 26.96 | 28.77 | 29.94 | 30.34 |
| 72 | 10.69 | 11.08 | 12.24 | 14.03 | 16.19 | 17.01 | 20.33 | 23.65 | 24.47 | 26.63 | 28.42 | 29.58 | 29.97 |
| 73 | 10.56 | 10.95 | 12.09 | 13.86 | 15.99 | 16.80 | 20.08 | 23.35 | 24.16 | 26.30 | 28.06 | 29.21 | 29.59 |
| 74 | 10.43 | 10.81 | 11.94 | 13.68 | 15.79 | 16.59 | 19.83 | 23.06 | 23.86 | 25.97 | 27.71 | 28.84 | 29.22 |
| 75 | 10.30 | 10.67 | 11.79 | 13.51 | 15.59 | 16.38 | 19.57 | 22.77 | 23.56 | 25.64 | 27.36 | 28.48 | 28.85 |
| 76 | 10.16 | 10.53 | 11.64 | 13.33 | 15.39 | 16.17 | 19.32 | 22.47 | 23.26 | 25.31 | 27.01 | 28.11 | 28.48 |
| 77 | 10.03 | 10.40 | 11.48 | 13.16 | 15.19 | 15.96 | 19.07 | 22.18 | 22.95 | 24.98 | 26.66 | 27.75 | 28.11 |
| 78 | 9.90 | 10.26 | 11.33 | 12.99 | 14.99 | 15.75 | 18.82 | 21.89 | 22.65 | 24.65 | 26.31 | 27.38 | 27.74 |
| 79 | 9.77 | 10.12 | 11.18 | 12.81 | 14.79 | 15.54 | 18.57 | 21.60 | 22.35 | 24.32 | 25.95 | 27.01 | 27.37 |
| 80 | 9.63 | 9.98 | 11.03 | 12.64 | 14.59 | 15.33 | 18.32 | 21.30 | 22.04 | 23.99 | 25.60 | 26.65 | 27.00 |
| 81 | 9.50 | 9.85 | 10.88 | 12.47 | 14.39 | 15.12 | 18.06 | 21.01 | 21.74 | 23.66 | 25.25 | 26.28 | 26.63 |
| 82 | 9.37 | 9.71 | 10.73 | 12.29 | 14.19 | 14.91 | 17.81 | 20.72 | 21.44 | 23.33 | 24.90 | 25.91 | 26.25 |
| 83 | 9.24 | 9.57 | 10.57 | 12.12 | 13.99 | 14.70 | 17.56 | 20.42 | 21.13 | 23.00 | 24.55 | 25.55 | 25.88 |
| 84 | 9.10 | 9.44 | 10.42 | 11.94 | 13.79 | 14.49 | 17.31 | 20.13 | 20.83 | 22.67 | 24.19 | 25.18 | 25.51 |
| 85 | 8.97 | 9.30 | 10.27 | 11.77 | 13.58 | 14.27 | 17.06 | 19.84 | 20.53 | 22.34 | 23.84 | 24.81 | 25.14 |
| 86 | 8.84 | 9.16 | 10.12 | 11.60 | 13.38 | 14.06 | 16.80 | 19.55 | 20.23 | 22.01 | 23.49 | 24.45 | 24.77 |
| 87 | 8.71 | 9.02 | 9.97 | 11.42 | 13.18 | 13.85 | 16.55 | 19.25 | 19.92 | 21.68 | 23.14 | 24.08 | 24.40 |
| 88 | 8.57 | 8.89 | 9.82 | 11.25 | 12.98 | 13.64 | 16.30 | 18.96 | 19.62 | 21.35 | 22.79 | 23.72 | 24.03 |
| 89 | 8.44 | 8.75 | 9.67 | 11.08 | 12.78 | 13.43 | 16.05 | 18.67 | 19.32 | 21.02 | 22.43 | 23.35 | 23.66 |
| 90 | 8.31 | 8.61 | 9.51 | 10.90 | 12.58 | 13.22 | 15.80 | 18.37 | 19.01 | 20.69 | 22.08 | 22.98 | 23.29 |
| 91 | 8.18 | 8.47 | 9.36 | 10.73 | 12.38 | 13.01 | 15.55 | 18.08 | 18.71 | 20.36 | 21.73 | 22.62 | 22.91 |
| 92 | 8.04 | 8.34 | 9.21 | 10.55 | 12.18 | 12.80 | 15.29 | 17.79 | 18.41 | 20.03 | 21.38 | 22.25 | 22.54 |
| 93 | 7.91 | 8.20 | 9.06 | 10.38 | 11.98 | 12.59 | 15.04 | 17.50 | 18.10 | 19.70 | 21.03 | 21.88 | 22.17 |
| 94 | 7.78 | 8.06 | 8.91 | 10.21 | 11.78 | 12.38 | 14.79 | 17.20 | 17.80 | 19.37 | 20.67 | 21.52 | 21.80 |
| 95 | 7.65 | 7.93 | 8.76 | 10.03 | 11.58 | 12.17 | 14.54 | 16.91 | 17.50 | 19.05 | 20.32 | 21.15 | 21.43 |
