# Supplementary material for: Normative reference values of handgrip strength for Brazilian older people aged 65 to 90 years: Evidence from the multicenter Fibra‑BR study
Source: PLoS One. 2021 May 4;16(5):e0250925. doi: 10.1371/journal.pone.0250925 (PMC8096087; doi:10.1371/journal.pone.0250925)
Supplement: S6 Table — (DOCX) [file pone.0250925.s016.docx]

# **S6 Table. Hand grip strength (*kgf*) projected for female ≤1.5 meters for a wide array of centiles.**

| **Age** | **Centiles for HGS (kgf)** | | | | | | | | | | | | |
| --- | --- | --- | --- | --- | --- | --- | --- | --- | --- | --- | --- | --- | --- |
|  | **2.5** | **3** | **5** | **10** | **20** | **25** | **50** | **75** | **80** | **90** | **95** | **97** | **97.5** |
| 65 | 10.41 | 10.78 | 11.87 | 13.54 | 15.57 | 16.35 | 19.46 | 22.57 | 23.34 | 25.37 | 27.05 | 28.14 | 28.51 |
| 66 | 10.34 | 10.70 | 11.78 | 13.45 | 15.46 | 16.23 | 19.32 | 22.41 | 23.18 | 25.19 | 26.86 | 27.94 | 28.30 |
| 67 | 10.26 | 10.62 | 11.70 | 13.35 | 15.35 | 16.11 | 19.18 | 22.25 | 23.01 | 25.01 | 26.66 | 27.74 | 28.10 |
| 68 | 10.19 | 10.55 | 11.61 | 13.25 | 15.24 | 15.99 | 19.04 | 22.09 | 22.84 | 24.83 | 26.47 | 27.54 | 27.89 |
| 69 | 10.11 | 10.47 | 11.53 | 13.15 | 15.13 | 15.88 | 18.90 | 21.93 | 22.67 | 24.65 | 26.28 | 27.33 | 27.69 |
| 70 | 10.04 | 10.39 | 11.44 | 13.06 | 15.02 | 15.76 | 18.76 | 21.76 | 22.51 | 24.47 | 26.08 | 27.13 | 27.49 |
| 71 | 9.96 | 10.31 | 11.36 | 12.96 | 14.90 | 15.64 | 18.62 | 21.60 | 22.34 | 24.28 | 25.89 | 26.93 | 27.28 |
| 72 | 9.89 | 10.24 | 11.27 | 12.86 | 14.79 | 15.53 | 18.48 | 21.44 | 22.17 | 24.10 | 25.70 | 26.73 | 27.08 |
| 73 | 9.81 | 10.16 | 11.19 | 12.77 | 14.68 | 15.41 | 18.34 | 21.28 | 22.01 | 23.92 | 25.50 | 26.53 | 26.87 |
| 74 | 9.74 | 10.08 | 11.10 | 12.67 | 14.57 | 15.29 | 18.20 | 21.12 | 21.84 | 23.74 | 25.31 | 26.33 | 26.67 |
| 75 | 9.67 | 10.00 | 11.02 | 12.57 | 14.46 | 15.17 | 18.06 | 20.96 | 21.67 | 23.56 | 25.11 | 26.13 | 26.46 |
| 76 | 9.59 | 9.93 | 10.93 | 12.48 | 14.35 | 15.06 | 17.93 | 20.79 | 21.50 | 23.38 | 24.92 | 25.92 | 26.26 |
| 77 | 9.52 | 9.85 | 10.85 | 12.38 | 14.23 | 14.94 | 17.79 | 20.63 | 21.34 | 23.19 | 24.73 | 25.72 | 26.06 |
| 78 | 9.44 | 9.77 | 10.76 | 12.28 | 14.12 | 14.82 | 17.65 | 20.47 | 21.17 | 23.01 | 24.53 | 25.52 | 25.85 |
| 79 | 9.37 | 9.70 | 10.68 | 12.18 | 14.01 | 14.71 | 17.51 | 20.31 | 21.00 | 22.83 | 24.34 | 25.32 | 25.65 |
| 80 | 9.29 | 9.62 | 10.59 | 12.09 | 13.90 | 14.59 | 17.37 | 20.15 | 20.84 | 22.65 | 24.14 | 25.12 | 25.44 |
| 81 | 9.22 | 9.54 | 10.51 | 11.99 | 13.79 | 14.47 | 17.23 | 19.99 | 20.67 | 22.47 | 23.95 | 24.92 | 25.24 |
| 82 | 9.14 | 9.46 | 10.42 | 11.89 | 13.68 | 14.35 | 17.09 | 19.82 | 20.50 | 22.28 | 23.76 | 24.71 | 25.03 |
| 83 | 9.07 | 9.39 | 10.34 | 11.80 | 13.57 | 14.24 | 16.95 | 19.66 | 20.33 | 22.10 | 23.56 | 24.51 | 24.83 |
| 84 | 8.99 | 9.31 | 10.25 | 11.70 | 13.45 | 14.12 | 16.81 | 19.50 | 20.17 | 21.92 | 23.37 | 24.31 | 24.63 |
| 85 | 8.92 | 9.23 | 10.17 | 11.60 | 13.34 | 14.00 | 16.67 | 19.34 | 20.00 | 21.74 | 23.18 | 24.11 | 24.42 |
| 86 | 8.84 | 9.16 | 10.08 | 11.51 | 13.23 | 13.89 | 16.53 | 19.18 | 19.83 | 21.56 | 22.98 | 23.91 | 24.22 |
| 87 | 8.77 | 9.08 | 10.00 | 11.41 | 13.12 | 13.77 | 16.39 | 19.02 | 19.66 | 21.38 | 22.79 | 23.71 | 24.01 |
| 88 | 8.70 | 9.00 | 9.91 | 11.31 | 13.01 | 13.65 | 16.25 | 18.85 | 19.50 | 21.19 | 22.59 | 23.50 | 23.81 |
| 89 | 8.62 | 8.92 | 9.83 | 11.21 | 12.90 | 13.54 | 16.11 | 18.69 | 19.33 | 21.01 | 22.40 | 23.30 | 23.61 |
| 90 | 8.55 | 8.85 | 9.74 | 11.12 | 12.78 | 13.42 | 15.97 | 18.53 | 19.16 | 20.83 | 22.21 | 23.10 | 23.40 |
| 91 | 8.47 | 8.77 | 9.66 | 11.02 | 12.67 | 13.30 | 15.83 | 18.37 | 19.00 | 20.65 | 22.01 | 22.90 | 23.20 |
| 92 | 8.40 | 8.69 | 9.57 | 10.92 | 12.56 | 13.18 | 15.70 | 18.21 | 18.83 | 20.47 | 21.82 | 22.70 | 22.99 |
| 93 | 8.32 | 8.62 | 9.49 | 10.83 | 12.45 | 13.07 | 15.56 | 18.04 | 18.66 | 20.29 | 21.63 | 22.50 | 22.79 |
| 94 | 8.25 | 8.54 | 9.40 | 10.73 | 12.34 | 12.95 | 15.42 | 17.88 | 18.49 | 20.10 | 21.43 | 22.29 | 22.58 |
| 95 | 8.17 | 8.46 | 9.32 | 10.63 | 12.23 | 12.83 | 15.28 | 17.72 | 18.33 | 19.92 | 21.24 | 22.09 | 22.38 |
