# Supplementary material for: Normative reference values of handgrip strength for Brazilian older people aged 65 to 90 years: Evidence from the multicenter Fibra‑BR study
Source: PLoS One. 2021 May 4;16(5):e0250925. doi: 10.1371/journal.pone.0250925 (PMC8096087; doi:10.1371/journal.pone.0250925)
Supplement: Attachment — Submitted filename: Response to Reviewers.docx [file pone.0250925.s017.docx]

Dear editor,

We appreciate the reviewers' consideration in pointing out adjustments in our work.

As requested, below are the answers and modifications to the raised points:

Comments from the editor

1. Please ensure that your manuscript meets PLOS ONE's style requirements, including those for file naming.
   a. All adjustments were made.
2. Please include in your methods section, the full names of the four ethics committees that reviewed and approved this study.
   a. Information added (lines 122-125, page 7.
3. We note that the grant information you provided in the ‘Funding Information’ and ‘Financial Disclosure’ sections do not match.
   a. This information has been corrected in the cover letter as well as in the article. The correct grant numbers have been entered in the “funding information” section (line 387).
4. We note you have included a table to which you do not refer in the text of your manuscript. Please ensure that you refer to Table 1, 2 and 4 in your text; if accepted, production will need this reference to link the reader to the Table.
   a. All four tables were cited in the first version of the manuscript (lines in 226, 227,250 and 295). In the current version, allusions in the text to Tables 1 to 4 are now in line.
5. Please upload a copy of Supporting Information Table S1, S2, S3, S4, S5 and S6 which you refer to in your text on page 27.
   a. This information was entered in the specific field.
6. Sample validation
   a. Adjustment was done. The sample validation was compounded by older people with low cognitive performance

Comments from reviewers

Reviewer#1:

1. Change the word 'subjects' to participants.
   a. Modified.
2. Line 301 change the word 'elderly' to older people/persons.
   a. Modified.
3. Pls add if HGS measurement followed standard procedures recommended by the American Society of Hand Therapists (ASHT).
   a. Information added (line 161, page 8).
4. Please add a section in discussion of if the NRV HGS results were comparable to other previous results.
   a. Information added (line 312, page 17 to line 318, page 18).

Reviewer #2: In line 221, in Table 1, there are inconsistencies that need to be clarified:
1. The numbers in some of the characteristics of the total sample are different, it is important to specify why at the footer. The numbers of the sum of the reference sample and the validation sample do not correspond in all the cases with 3128, and 2447, respectively.

a. We point this out in the footer of Table 1. Please note that the variables of interest (sex, age, height and HGS) do not hold missing data.

3. The percentages of the total sample are calculated per column, while the percentages in the reference sample and in the validation sample, are not clear how they were calculated.

a. All adjustments were made.
